# Methods used in 'ALDH2*2 association with longevity and reduced risk of cognitive decline in Japanese-American men'

**DOI:** 10.1093/geroni/igab046.2515

**Published:** 2021-12-17

**Authors:** Anubhav Nikunj Singh Sachan, Steven Edland, Julia Chosy, Lenore Launer, Sudha Seshadri, Lon White

**Affiliations:** 1 University of California San Diego, La Jolla, California, United States; 2 Pacific Health Research and Education Institute, Honolulu, Hawaii, United States; 3 National Institute on Aging, Bethesda, Maryland, United States; 4 UT Health San Antonio, San Antonio, Texas, United States

## Abstract

ALDH2*2 is a loss of function mutation common in East Asian populations associated with facial flushing on exposure to alcohol and increased risk of certain cancers. Conversely, absence of the ALDH2*2 mutation is associated with increased risk of hypertension and cerebral microbleeds, and two recent studies report a higher frequency of ALDH2*2 alleles in nonagenarians compared to population control samples. We used survival analysis to investigate the association between ALDH2*2 and risk of cognitive impairment and death after controlling for midlife alcohol consumption and other covariates. Participants are 621 Japanese-American men (72 to 92) enrolled in the Honolulu Asia Aging Study (HAAS) and assessed for cognitive impairment for up to 20 years. Impairment was defined as crossing below a threshold score of 74 on the Cognitive Assessment Screening Instrument (CASI). Age at death was determined by Hawaii state death certificate. Ounces of ethyl alcohol consumed per month was assessed by structured interview (number, frequency, and type of beverage) conducted 25 years prior to baseline cognitive assessment. Persons heterozygous for the ALDH2*2 variant have reduced risk of cognitive impairment and reduced risk of death, compared to homozygote non-carriers. Covarying by alcohol exposure had no effect on observed associations. This study replicates previous findings associating ALDH2*2 with longevity, and provides evidence the protective effect extends to cognition. This poster details the statistical analysis carried out to obtain these results.

